# Metabolic remodeling of endometriosis microenvironment: Energy stress and immune evasion

**DOI:** 10.1016/j.isci.2026.116592

**Published:** 2026-07-09

**Authors:** Pusheng Yang, Tao Wang, Wenwen Liu, Yaxin Miao, Yiping Zhu, Jing Sun

**Affiliations:** 1 Shanghai Key Laboratory of Maternal Fetal Medicine, Shanghai Institute of Maternal-Fetal Medicine and Gynecologic Oncology, Shanghai First Maternity and Infant Hospital, School of Medicine, Tongji University, Shanghai 200092, China

**Keywords:** endometriosis, metabolic reprogramming, energy Stress, immune evasion

## Abstract

Endometriosis (EMs) is an estrogen-dependent chronic inflammatory gynecological disease characterized by ectopic growth of endometrial tissues, leading to dysmenorrhea, pelvic pain, and infertility. Although the retrograde menstruation theory clarifies the dissemination of endometrial fragments to ectopic sites, the mechanisms behind the survival of ectopic lesions and their immune evasion in hostile microenvironments remain unclear. Endometrial stromal cells (ESCs) are chronically exposed to a microenvironment of hypoxia, nutrient deprivation and oxidative stress, and this energy stress state drives the ESCs to develop adaptive metabolic reprogramming. Through remodeling glucose, lipid, and amino acid metabolic pathways, ESCs not only fulfill their own proliferative requirements but also utilize metabolites as signaling mediators to modulate immune cell functions. This review elaborates on the characteristics of energy stress-driven metabolic reprogramming in EMs, deciphers its mechanisms underlying immune evasion, and discusses the therapeutic potential of combined metabolic-immune intervention strategies.

## Graphical abstract

**Figure:**
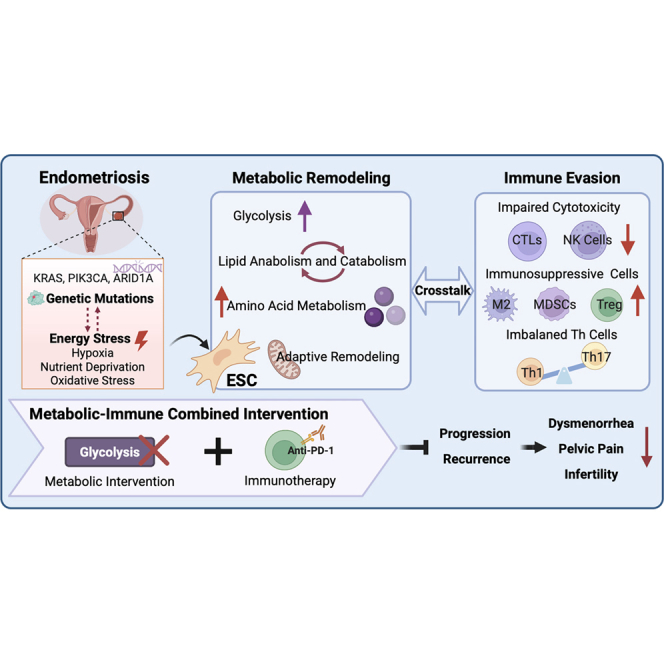

## Teaser

Physiology; Molecular biology; Cell biology

## Background

Endometriosis (EMs) is an estrogen-dependent chronic inflammatory disease characterized by the growth and survival of viable endometrial glands and stroma outside the uterine cavity, often causing dysmenorrhea, pelvic pain, and infertility.^1^^,^^2^ As a prevalent gynecological disorder, EMs impacts 10%–15% of women of reproductive age, with up to 50% of infertile women diagnosed with the condition.^3^ Due to the side effects of hormonal therapy, the risks and high recurrence rates linked to surgery, and the need for fertility preservation, the clinical management of EMs remains a considerable challenge, severely affecting patients’ physical and mental health as well as their quality of life.^4^^,^^5^ Although the widely accepted retrograde menstruation theory provides insights into the dissemination of endometrial fragments to ectopic sites, the mechanisms underlying the survival, proliferation, and invasion of endometriotic lesions in the ectopic microenvironment remain incompletely elucidated.

Accumulating evidence indicates that immune escape arising from defective immune surveillance and immune dysfunction acts as a critical factor of endometriotic lesion progression.^6^ Under physiological conditions, retrograde endometrial fragments are recognized and eliminated by the immune system, whereas a dysfunctional immune microenvironment is observed at endometriotic lesion sites.^7^^,^^8^ Cytotoxic T lymphocytes (CTLs) and natural killer (NK) cells exhibit impaired functions, accompanied by an imbalanced ratio of T helper (Th) cells, as well as extensive infiltration of immunosuppressive cell populations including M2-type macrophages, regulatory T cells (Tregs), and myeloid-derived suppressor cells (MDSCs).^9^^,^^10^ This unique immunosuppressive microenvironment allows ectopic lesions to achieve immune escape, thereby sustaining their survival and progression. However, the specific driving factors underlying this process remain to be further explored.^11^^,^^12^

As a core mechanism for cellular adaptation to microenvironmental changes, metabolic reprogramming has been well characterized in cancer, infectious diseases, and autoimmune disorders.^13^^,^^14^^,^^15^ Although EMs is a benign disorder, it exhibits multiple cancer-like malignant biological behaviors, including abnormal proliferation, adhesion, invasion, and neovascularization.^16^ Accumulating studies have indicated that endometrial stromal cells (ESCs) reside in an energy stress microenvironment characterized by intermittent hypoxia, nutrient deprivation, and sustained oxidative stress.^17^^,^^18^ To survive in such an adverse microenvironment, ESCs make adaptive adjustments via increased glucose uptake, upregulated glycolysis, enhanced lipid uptake and utilization, and modified amino acid metabolism, which not only provide necessary substrates for proliferation and invasion but also enable metabolic products to function as signaling molecules regulating immune cell activities, thus facilitating immune escape.^19^^,^^20^ Thus, metabolic reprogramming is a crucial driver of immune escape and EMs progression.

This review seeks to emphasize the central status of energy stress, systematically clarify the metabolic characteristics of the EMs microenvironment, and elucidate its molecular mechanisms in promoting EMs progression by mediating immune escape via the modulation of immune cell phenotypic differentiation and functional activity. This is the first study to construct a comprehensive energy stress-metabolic reprogramming-immune evasion regulatory network in EMs. Furthermore, in contrast to previous reviews that focus on single-target therapeutic strategies, this review elaborates on the therapeutic potential of metabolic-immune combined intervention strategies targeting the energy stress regulatory axis, which provides a novel and comprehensive theoretical foundation as well as valuable research insights for developing innovative therapeutic strategies for EMs.

## Imbalance and regulation of energy stress in EMs

The adverse ectopic microenvironment, characterized by hypoxia, nutritional deprivation, and oxidative stress, serves as the core driver inducing ESCs to enter a state of persistent energy stress and initiates subsequent metabolic adaptation.^21^ During the pathological progression of EMs, endometrial tissues that detach from the stable intrauterine environment and implant at ectopic sites are subjected to local hypoxia driven by multiple factors, including inherent hypoperfusion, a sharp increase in oxygen consumption caused by the active proliferation of ESCs, delayed formation and impaired function of neovascularization, and vascular compression by local lesion fibrosis.^22^^,^^23^^,^^24^ The inherently nutrient-deficient peritoneal cavity cannot satisfy the demands for glucose, nucleotides, and adenosine triphosphate (ATP) required for the active proliferation of ESCs, resulting in nutritional deprivation and supply-demand imbalance.^25^ Meanwhile, hypoxia, chronic inflammation, and iron overload induced by periodic lesion hemorrhage generate excessive reactive oxygen species (ROS), which induce mitochondrial membrane lipid peroxidation, mitochondrial DNA damage, and electron transport chain complex dysfunction, leading to decreased oxidative phosphorylation (OXPHOS) efficiency, insufficient ATP synthesis, and ultimately causing oxidative stress and energy deficit.^26^^,^^27^ Together, these stressors establish a chronic energy stress state that promotes extensive metabolic reprogramming in ESCs to support their survival and progression.

### Metabolic characteristics of energy stress in EMs

#### Enhanced glycolysis

Under energy stress, ectopic lesions undergo adaptive alterations in their metabolic phenotypes, characterized by increased glucose uptake, upregulated glycolysis, elevated lactate accumulation, and impaired mitochondrial function.^28^^,^^29^ Studies have shown that glucose transporter 1, 3, and 4 (GLUT1, GLUT3, and GLUT4) have significantly higher mRNA and protein levels in ectopic lesions compared to normal endometrial tissues, which substantially improves cellular glucose uptake efficiency and provides a material basis for high-intensity energy metabolism.^18^^,^^30^ Glucose-6-phosphate, an intermediate product of glycolysis, can be diverted into the pentose phosphate pathway (PPP), and abundant nicotinamide adenine dinucleotide phosphate (NADPH) generated thereby enhances antioxidant capacity to resist oxidative damage. Meanwhile, its metabolic products provide precursors for nucleotide and lipid synthesis, thereby supporting the active proliferation of ESCs.^31^ In addition, extensive lactate accumulation derived from enhanced aerobic glycolysis can further promote cell invasiveness and induce a local acidic microenvironment that impairs immune cell function.^17^^,^^32^

#### Dysregulation of lipid anabolism and catabolism

EMs has been recognized to be associated with aberrant lipid metabolism.^33^^,^^34^ Peroxisome proliferator-activated receptors (PPARs) are activated, and this upregulates the expression of lipoprotein lipase (LPL) in ESCs, leading to a marked increase in exogenous lipid uptake.^35^ Meanwhile, stimulated fatty acid synthesis pathways and upregulated fatty acid synthase (FASN) in ESCs accelerate the conversion of acetyl-CoA to fatty acids to support rapid cell proliferation during energy stress.^35^^,^^36^ In addition, enhanced activity of phospholipid synthesis enzymes like sphingomyelin synthase (SMS) drives the production of phosphatidylcholine, satisfying the membrane structural requirements of rapidly proliferating ESCs.^37^^,^^38^ Notably, large accumulations of lipids and their metabolic intermediates can also activate inflammatory signaling pathways, disrupt redox homeostasis, and exacerbate local chronic inflammation, thereby accelerating the progression of EMs.^39^^,^^40^

#### Reprogramming of amino acid metabolism

ESCs have been reported to reprogram amino acid metabolism to adapt to the energy stress of ectopic lesions, with key characteristics including enhanced amino uptake, metabolic hyperactivity, and metabolic products-mediated immune evasion.^41^^,^^42^^,^^43^ To enhance glutamine uptake, ESCs upregulate alanine, serine, cysteine transporter 2 (ASCT2, also known as SLC1A5). Highly expressed glutaminase 1 (GLS1) then catalyzes the conversion of intracellular glutamine into glutamate, which is either used as a precursor for the antioxidant glutathione (GSH) to reduce oxidative stress or converted to α-ketoglutarate (α-KG) to fuel the tricarboxylic acid (TCA) cycle.^44^^,^^45^ Studies have demonstrated that the serine-glycine metabolic pathway is also markedly upregulated in EMs by chronic stress. This pathway provides one-carbon units for ESC proliferation, essential substrates for collagen synthesis to improve cell adhesion and invasion, and reducing equivalents that scavenge ROS and maintain cellular redox homeostasis.^46^ More importantly, indoleamine 2,3-dioxygenase-1 (IDO1) and tryptophan 2,3-dioxygenase (TDO) have been reported to be significantly overexpressed in ectopic lesions.^47^^,^^48^^,^^49^ As key enzymes in tryptophan catabolism, they effectively catalyze the degradation of tryptophan via the kynurenine metabolic pathway, which markedly reduces tryptophan levels in the local microenvironment of ectopic lesions. Tryptophan is a necessary amino acid for T cell activation and proliferation, and its depletion induces CTL dysfunction and impaired immune responses. And its metabolites further promote Treg differentiation and the establishment of a local immune-tolerant microenvironment, which facilitates immune evasion of ectopic lesions and accelerate the progression of EMs.^50^^,^^51^^,^^52^

#### Mitochondrial adaptive remodeling

Mitochondria serve as the core hub of energy metabolism, and their adaptive capacities including stress resistance, metabolic switching, and structural remodeling undergo remarkable alterations in response to energy stress, which consequently acts as a key pathological driver of the survival, invasion and microenvironmental perturbation of ectopic lesions.^53^^,^^54^ Mitochondrial adaptive defects are mainly manifested as imbalanced mitochondrial dynamics and mitochondrial DNA (mtDNA) alterations, both of which trigger mitochondrial dysfunction and facilitate adaptive metabolic reprogramming.^55^^,^^56^ Mitochondrial cristae remodeling, a typical feature of imbalanced mitochondrial dynamics, is closely associated with the functional regulation of ESCs. Experimental studies have confirmed that ectopic lesions show markedly reduced expression of the mitochondrial fusion-related proteins, such as mitofusin 1 (MFN1), mitofusin 2 (MFN2), and optic atrophy 1 (OPA1), leading mitochondria to exhibit more lamellar cristae and wider cristae cavities.^57^ This structural alteration of cristae has been confirmed to be associated with enhanced mitochondrial respiratory capacity, and we hypothesize it may alleviate oxidative injury and promote the survival of ectopic ESCs.^58^ Another study has observed that short rod-shaped or granular mitochondria in ESCs, which are accompanied by partial impairment of mitochondrial function, yet such abnormalities do not result in significant changes in cell survival rates.^59^ This phenomenon is supported by experimental evidence and may be attributed to the effective adaptive compensation of ESCs via subsequent metabolic reprogramming. Drawing on existing experimental evidence and advances in mitochondrial research, we hypothesize that mitochondrial adaptive remodeling of cristae structure may modulate the spatial assembly of mitochondrial respiratory chain complexes, which further enhances dependence on alternative metabolic pathways to compensate for insufficient mitochondrial OXPHOS energy supply, alleviate oxidative damage caused by energy stress, improve the overall mitochondrial energy supply efficiency, and ultimately promote the survival and migration of ESCs.^57^^,^^60^^,^^61^^,^^62^

Notably, mtDNA alterations are another crucial cause of mitochondrial dysfunction in ESCs under energy stress, and such alterations are manifested as unchanged copy number but significant mutations and structural abnormalities, which form a direct causal link with the decline of mitochondrial OXPHOS capacity supported by experimental data.^63^^,^^64^ Studies have reported that mtDNA copy number in ectopic ESCs shows no significant difference from that in normal and eutopic ESCs, suggesting that ESCs do not rely on increasing mtDNA replication to compensate for mitochondrial function under energy stress. However, energy stress can induce obvious mtDNA mutations and deletions in ESCs (such as D-loop region polymorphisms and the mtDNA T16189C variant) and typical mtDNA ultrastructure abnormalities (including asymmetric electron-lucent swollen areas without cristae on one side and circular cristae with separated inner and outer membranes).^63^^,^^64^^,^^65^^,^^66^ These mtDNA mutations and structural abnormalities may lead to assembly failure and dysfunction of electron transport chain complexes, thereby representing vital contributors to mitochondrial OXPHOS deficiency and decreased ATP synthesis efficiency in ESCs.^67^^,^^68^ Additionally, to adapt to persistent energy stress in ectopic lesions, ESCs exhibit significantly enhanced mitochondrial autophagy, which selectively eliminates damaged mitochondria, thereby restoring mitochondrial membrane potential, maintaining mitochondrial functional stability, and driving metabolic reprogramming.^69^^,^^70^^,^^71^ Studies have demonstrated that mammalian STE20-like kinase 1 (Mst1) suppresses Parkin-dependent mitophagy through P53 modulation, leading to impaired mitophagy and subsequent cellular oxidative stress, calcium overload as well as metabolic dysfunction.^72^^,^^73^^,^^74^ A study confirmed that Mst1 expression is markedly downregulated in ectopic lesions, which inhibits apoptosis and enhances migration of ESCs by regulating DRP1-associated mitochondrial fission and Parkin-dependent mitophagy, thus forming a protective mechanism for ESCs to survive under the condition of mitochondrial dysfunction induced by mtDNA alterations and cristae structural imbalance.^75^

### Adaptive mechanisms of EMs under combined drive of genetic mutation and energy stress

#### Genetic mutation-mediated metabolic reprogramming in EMs

Genetic mutations and metabolism alterations are recognized as hallmark characteristics of cancer.^76^^,^^77^ During tumorigenesis, somatic alterations in oncogenes trigger extensive transcriptional changes and consequently elicit robust metabolic reprogramming.^78^ Mounting evidence indicates that genetic and metabolic signals interact continuously within the tumor microenvironment and together drive metabolic reprogramming in tumor cells. Ying et al. demonstrated that oncogenic KRAS (G12D) maintains pancreatic tumors by stimulating glucose uptake and glycolysis.^79^ Kerr et al. reported that increased copy number of mutant KRAS can reprogram the metabolism of lung cancer cells, converting them from a highly glycolytic state to one with elevated Krebs cycle and glutathione synthesis capacity, thereby promoting tumor cell invasiveness.^80^ Moreover, Hao et al. demonstrated that oncogenic PIK3CA mutations cause glutamine dependency in colorectal cancer, characterized by the conversion of significantly more glutamine into α-ketoglutarate to fuel the tricarboxylic acid cycle and generate ATP.^81^ Notably, in a landmark genomic study, Anglesio et al. identified recurrent somatic mutations in canonical cancer driver genes including KRAS, PIK3CA, and ARID1A in approximately 26% of deep infiltrating endometriotic lesions, even in the absence of malignant transformation. These mutations are highly enriched in epithelial cells and cf. strong selective advantages, strongly supporting their role as intrinsic drivers of EMs initiation and progression.^82^ However, there may be limited prior research on the relationship between cancer-related gene mutations and metabolic reprogramming in EMs. By drawing on well-established tumor metabolic mechanisms, we hypothesize an integrated dual-driver model in which genetic mutations function as upstream initiators of active metabolic reprogramming, while energy stress acts as a parallel and synergistic extrinsic amplifier that further stabilizes and reinforces pathological metabolic phenotypes of EMs. Further investigation is required to validate this hypothesis in future studies.

#### Energy stress-induced metabolic adaptation in EMs

##### Activation of hypoxia inducible factor-1α (HIF-1α) and its regulatory network

**Figure 1:**
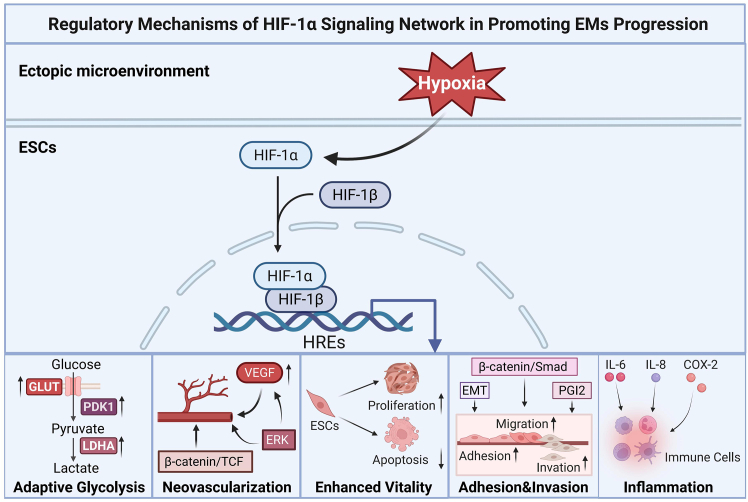
Activation of HIF-1α and its regulatory network in the EMs energy stress microenvironment Hypoxia in ectopic lesions induces HIF-1α upregulation and nuclear translocation, which combines with HIF-1β to initiate target gene transcription via HREs and thus promote EMs progression through multiple pathways: (1) upregulating key glycolytic enzymes including GLUT, PDK1, and LDHA to reshape glycolysis; (2) activating VEGF and the β-catenin/TCF and ERK pathways to induce angiogenesis; (3) regulating CCND1 and BNIP3 to inhibit apoptosis and enhance cell survival; (4) promoting the adhesion and migration of ESCs via the β-catenin/Smad pathway, EMT and PGI2; (5) inducing the release of inflammatory factors such as IL-6 and IL-8 to amplify the inflammatory response.

Energy stress-induced hypoxia in the ectopic microenvironment acts as the primary and core upstream trigger for HIF-1α pathway activation. HIF-1 is a heterodimer consisting of an oxygen-dependent labile α-subunit and a constitutively stable β-subunit.^83^ Hypoxia in ectopic lesions significantly upregulates, stabilizes, and induces nuclear translocation of HIF-1α, which heterodimerizes with HIF-1β and binds to hypoxia-responsive elements (HREs). This triggers target gene transcription, regulating processes such as glucose metabolism, angiogenesis, adhesion, and migration, as well as inflammatory responses, which in turn preserves the viability and regenerative capacity of ESCs under energy stress, thereby facilitating the progression of EMs.^84^^,^^85^^,^^86^ Specifically, upregulated HIF-1α in ectopic endometrium promotes the transcription of multiple key glycolytic enzymes including GLUTs, pyruvate dehydrogenase kinase 1 (PDK1) and lactate dehydrogenase A (LDHA), driving the metabolic remodeling of ESCs to adapt to the energy-stressed microenvironment.^87^^,^^88^ Angiogenesis represents a critical pathological process in EMs.^89^ HIF-1α can directly bind to HREs in the promoter region of vascular endothelial growth factor (VEGF) to activate its transcription. It can also enhance the expression of proangiogenic factors via the β-catenin/TCF and ERK signaling pathways, inducing neovascularization and facilitating the occurrence and development of EMs.^90^^,^^91^^,^^92^ HIF-1α is also reported to regulate cyclin D1 (CCND1) and BCL2 interacting protein 3 (BNIP3), thereby alleviating energy stress-induced apoptosis and enhancing cell survival.^93^ It can also enhance the adhesion and migration capacities of ESCs by modulating the β-catenin/Smad pathway, inducing epithelial-mesenchymal transition (EMT) process, and regulating prostaglandin I_2_ (PGI_2_) levels.^94^^,^^95^^,^^96^^,^^97^ In addition, highly expressed HIF-1α in ectopic lesions can stimulate the expression of interleukin-6 (IL-6), interleukin-8 (IL-8), cyclooxygenase-2 (COX-2) and high-mobility group box chromosomal protein 1 (HMGB1), inducing and maintaining local inflammatory responses which may be associated with the development of chronic pelvic pain in EMs patients.^98^^,^^99^ Notably, HIF-1α can interact extensively with NF-κB and other inflammatory pathways, leading to amplified inflammatory responses and the subsequent formation of a hypoxia-inflammation vicious cycle and ultimately exacerbating EMs progression (Figure 1).^93^^,^^100^

##### Lactic acid accumulation and PKM2-regulated acidic microenvironment formation

**Figure 2:**
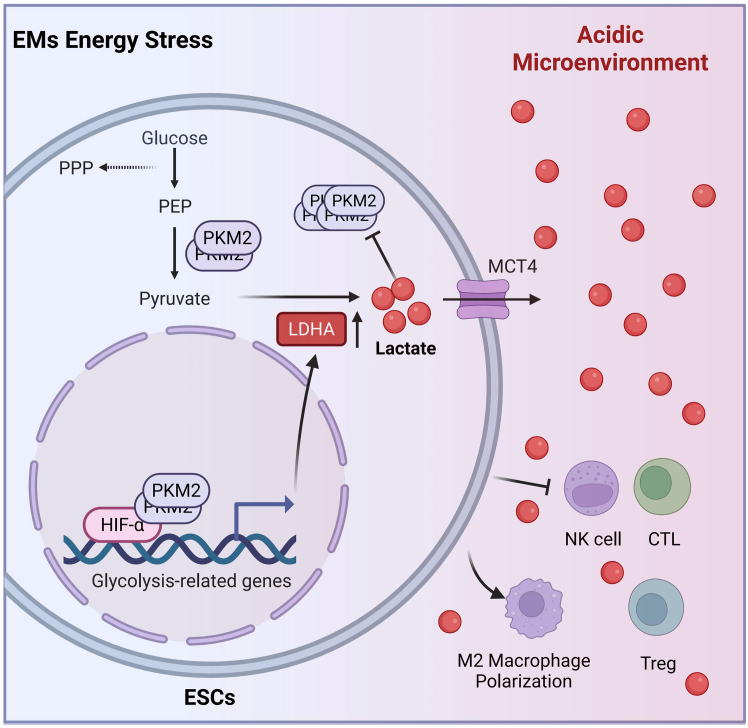
Lactic acid accumulation and PKM2 activation mediate acidic microenvironment formation to promote EMs progression HIF-1α upregulates LDHA and facilitates massive lactate production and efflux via MCT4, resulting in elevated lactate levels in ectopic lesions. Dimeric PKM2 reduces mitochondrial pyruvate entry to supply lactate precursors, translocates into the nucleus as a HIF-1α coactivator to upregulate glycolysis-related genes while the acidic microenvironment inhibits PKM2 conversion to tetramers. The ectopic acidic microenvironment suppresses the cytotoxic activity of NK cells and CTLs, promotes M2 macrophage polarization and Treg function, facilitates the immune escape of ESCs, and ultimately drives the progression of EMs.

Energy stress, characterized by hypoxia and glucose metabolic disorder in ectopic microenvironment, serves as the core upstream driver of pyruvate kinase M2 (PKM2) conformational change and lactic acid accumulation. Research has demonstrated that highly expressed HIF-1α in ectopic lesions significantly increases the expression of LDHA, which catalyzes the conversion of pyruvate to lactate and produces large amount of lactate. Local lactate levels in ectopic lesions are more than twice as high as those in normal endometria when combined with effective lactate efflux facilitated by highly expressed monocarboxylate transporter 4 (MCT4).^101^ The function of the terminal key enzyme in glycolysis, PKM2, is directly determined by its conformational state. The primary form of PKM2 under normal physiological conditions is a highly active tetramer that quickly catalyzes the conversion of phosphoenolpyruvate (PEP) to pyruvate, continuously depleting upstream glycolytic substrates and generating large amounts of pyruvate that enter the TCA cycle to fuel efficient aerobic cellular energy production. According to studies, despite being markedly upregulated in ESCs, PKM2 is predominantly a low-activity cytoplasmic dimer that significantly reduces the catalytic efficiency of PEP-to-pyruvate conversion. Glucose deprivation under energy stress further reinforces this tetramer-to-dimer switch of PKM2, which decreases pyruvate entry into mitochondria for aerobic metabolism in the hypoxic microenvironment of lesions, resulting in the build-up of glycolytic intermediates. Some of these intermediates are shunted into pathways such as the PPP to supply biosynthetic precursors (including nucleotides) and reducing equivalents for rapid cell proliferation. Meanwhile, it causes pyruvate to continuously build up, which provides an adequate substrate for the synthesis of lactate.^31^^,^^102^ Besides, dimeric PKM2 was reported to translocate into the nucleus to function as a coactivator of HIF-1α, increasing the transcription of multiple glycolysis-related genes, such as LDHA, and further exacerbating the development of an acidic microenvironment. Consequently, PKM2 remains primarily dimeric because the acidic microenvironment directly hinders the formation and stability of the highly active PKM2 tetramer, which creates a metabolic positive feedback loop that ultimately leads to the persistent and massive production of lactate within lesions.^18^^,^^30^^,^^87^ It has been reported that multiple matrix metalloproteinases (MMPs) can be activated by the acidic microenvironment in ectopic lesions, which greatly improves ESC migration and invasion capabilities and promotes angiogenesis through VEGF secretion.^103^ Notably, studies have shown that this acidic microenvironment directly suppresses the activities of NK cells and CTLs, and simultaneously induce M2 macrophage polarization and increases Treg function, creating an immunosuppressive niche that protects ESCs from immune elimination (Figure 2).^6^^,^^104^

##### Activation of the AMPK/mTOR energy sensing pathway

AMP-activated protein kinase (AMPK) and mammalian target of rapamycin (mTOR) together form the energy-sensing system of EMs. Energy stress dynamically regulates this system to drive the adaptive survival of ESCs.^105^^,^^106^ AMPK is a heterotrimeric serine/threonine protein kinase composed of α, β and γ subunits and maintains cellular energy homeostasis via sensing the intracellular AMP/ATP ratio.^107^^,^^108^ Under energy stress in EMs, mitochondrial adaptive defects in ESCs lead to decreased ATP production and an elevated intracellular AMP/ATP ratio, prompting AMP to directly and allosterically activate AMPK. Concurrently, energy stress-induced oxidative stress stimulates ROS accumulation from dysfunctional mitochondria, which further mediates AMPK activation via calcineurin (CaN) and synergistically amplifies the energy stress signal.^109^ Activated AMPK subsequently acts as an energy switch to maintain energy homeostasis by promoting ATP catabolism and inhibiting ATP-consuming pathways, and it suppresses mTOR activity through phosphorylation to form a negative feedback loop, thus cooperatively counteracting energy stress in EMs. Studies have demonstrated that AMPK significantly upregulates the expression of GLUT1, hexokinase 2 (HK2) and phosphofructokinase-2 (PFK2), enhances glycolysis and ensures energy supply.^110^ By inhibiting the mTOR pathway, AMPK also phosphorylates and activates unc-51 like autophagy activating kinase 1 (ULK1) to initiate autophagy, which degrades damaged mitochondria and abnormal proteins, and recycles energy metabolites for relieving energy stress-induced nutrient deprivation.^111^^,^^112^ Furthermore, the mTOR pathway modulates DNA and protein synthesis as well as cell cycle progression in ESCs, driving these cells to enter a low-metabolism survival mode to adapt to energy stress.^113^ Additionally, evidence indicates that AMPK decreases the levels of inflammatory cytokines including IL-8 and monocyte chemoattractant protein 1 (MCP-1) in ectopic lesions, and exerts anti-inflammatory activity via inhibition of the mTOR and NF-κB signaling pathways.^114^

##### Involvement of epigenetic regulation

**Figure 3:**
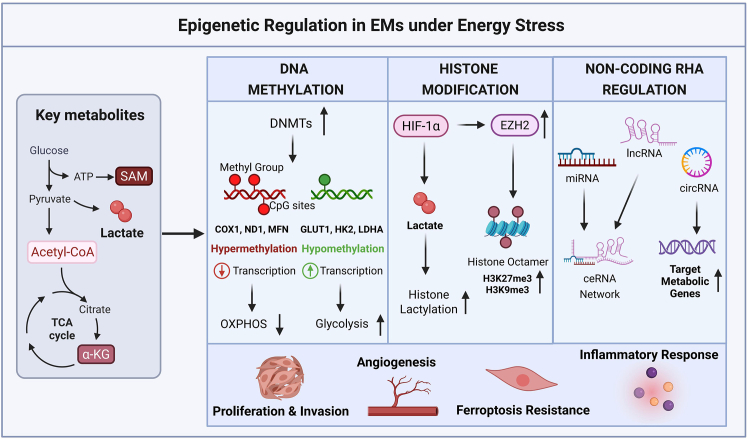
Epigenetic regulation under energy stress in EMs Energy stress in EMs modulates epigenetic processes (DNA methylation, histone modification, non-coding RNA regulation) via metabolic products to enable long-term cellular metabolic adaptation: (1) elevated DNMT drives glycolytic compensation; (2) HIF-1α regulates EZH2-mediated histone modification to stabilize ESC metabolic phenotypes; (3) non-coding RNAs target metabolic genes to complement epigenetic regulation.

Energy stress not only triggers the rapid activation of intracellular signaling pathways, but also causes epigenetic changes in ESCs, including DNA methylation, histone modification, and non-coding RNA regulation. These modifications steadily preserve the metabolic adaptive phenotype of ESCs without altering DNA sequences, thereby enabling long-term metabolic adaptation to energy stress.^115^ Key metabolites under energy stress in EMs, including acetyl-CoA, S-adenosylmethionine (SAM), α-KG, and lactic acid, serve as essential substrates or cofactors for epigenetic modifications, directly influencing the activity of epigenetic modifying enzymes and substrate availability.^116^^,^^117^ Studies have demonstrated that ectopic endometrial samples exhibit a marked upregulation of DNA methyltransferases (DNMTs), which results in altered methylation status in the promoter regions of genes related to energy metabolism. Specifically, significantly elevated methylation levels in the promoter regions of important OXPHOS genes, such as mitofusin (MFN), NADH dehydrogenase subunit 1 (ND1), and cytochrome oxidase subunit 1 (COX-1), inhibit transcription and reduce the efficiency of aerobic metabolism. On the other hand, ectopic ESCs are able to sustain compensatory glycolysis under energy stress for cell proliferation and migration demands due to hypomethylation in the promoters of important glycolytic genes such as GLUT1, HK2, and LDHA.^118^^,^^119^ Studies have also shown that the core enzymes mediating DNA demethylation, ten-eleven translocation methylcytosine dioxygenases (TETs), are significantly downregulated in ectopic lesions and modulate the adhesion and migration of ESCs via the EMT process.^120^^,^^121^ The histone methyltransferase enhancer of zeste 2 polycomb repressive complex 2 subunit (EZH2) is markedly upregulated in endometriotic lesions along with elevated levels of H3K27me3 and H3K9me3. HIF-1α controls this process to further stabilize the metabolic reprogramming phenotype and control ESC adhesion, migration, proliferation, and angiogenesis.^122^ Additionally, through the competing endogenous RNA (ceRNA) network, non-coding RNAs can directly target metabolism-related genes or modulate ESC proliferation, apoptosis, signal transduction, and inflammatory responses, serving as a vital supplement to epigenetic regulation under energy stress.^123^^,^^124^^,^^125^ Accumulation of lactic acid has also been shown to increase cellular resistance to ferroptosis by inducing lactylation of histone lysine residues.^101^ Notably, cancer-associated driver mutations such as KRAS, PIK3CA, and ARID1A act as intrinsic triggers and may conjointly shape the metabolic reprogramming landscape of EMs together with energy stress.^82^^,^^126^ Together, energy stress-induced epigenetic alterations and genetic mutations establish a direct link between metabolism and gene expression, forming an integrated regulatory network that mediates pathological metabolic reprogramming and drives the progression of ectopic lesions (Figure 3).

## Metabolic switches for immune evasion in EMs

EMs progression largely depends on the ability of ESCs to successfully evade immune surveillance and elimination.^20^ Studies have confirmed that this immune evasion process is not passive but arises from the dynamic interplay between ESCs and the immune system within a specific microenvironment.^127^^,^^128^ In the energy-stressed microenvironment, ectopic lesions actively modulate immune cell functions through active metabolic remodeling and reshape the immunosuppressive microenvironment, which acts as a key driver of EMs initiation and progression.^129^

### Metabolic paralysis of immune cell functions in EMs

#### Metabolic-driven polarization shift of macrophages

As the predominant cell type in ectopic lesions, macrophages are significantly increased in both the peritoneal fluid and ectopic lesions of EMs patients, whereas their phagocytic capacity is notably impaired.^130^^,^^131^ Studies show that M1 macrophages dominate the early stage of EMs, secreting massive pro-inflammatory cytokines and chemokines to trigger early tissue damage and inflammation. With disease progression and aggravated energy stress in the local microenvironment, macrophage polarization shifts toward the immunosuppressive, tissue-repairing M2 phenotype, promoting lesion progression, and immune evasion.^132^^,^^133^ It has been discovered that hypoxic microenvironment of ectopic lesions induces M2 macrophage polarization via upregulating the HIF-1α/Sema3A pathway, which promotes the proliferation and migration of ESCs.^134^ Hypoxia significantly increases the expression of the “don’t eat me” signal molecule cluster of differentiation 47 (CD47) in ectopic lesions, which not only inhibits macrophage phagocytic function but also triggers the CD47/AKT-LDHA glycolysis pathway to enhance lactate production and thus accelerates M2 macrophage polarization.^135^ Moreover, lactate accumulation from energy stress in ectopic lesions can also induce M2 macrophage polarization via the Mettl3/Trib1/ERK/STAT3 signaling pathway.^32^ Additionally, elevated HIF-1α in ectopic lesions directly upregulates COX-2, the key enzyme for prostaglandin E_2_ (PGE_2_) synthesis, while enhanced fatty acid uptake and synthesis under energy stress ensure an adequate supply of arachidonic acid, the PGE_2_ precursor, to synergistically fuel substantial PGE_2_ production. High PGE_2_ levels not only impair the phagocytic function of peritoneal macrophages by inhibiting CD36 but also suppress pro-inflammatory mediators such as inducible nitric oxide synthase (iNOS) and IL-12, while upregulating immunosuppressive and tissue repair factors including arginase-1 (ARG1), IL-10 and transforming growth factor β (TGF-β).^136^ This drives macrophage polarization toward the M2 phenotype, promotes tissue repair and fibrosis and ultimately exacerbates pelvic adhesion and chronic pain in EMs patients.^137^^,^^138^ Notably, abundant infiltrating M2 macrophages highly express ARG1 to mediate arginine deprivation, which impairs T cell immune responses and further aggravates the immunosuppressive microenvironment of ectopic lesions.^139^

#### Metabolic restriction of NK cell cytotoxicity

As key innate immune effector cells, NK cells are supposed to perform immune surveillance via recognizing and clearing ESCs. However, studies have shown that the energy-stressed microenvironment of ectopic lesions induces abnormal NK cell function with markedly restricted cytotoxicity.^140^ The activation and cytotoxicity of NK cells are highly dependent on energy supply from glucose metabolism.^141^ In the nutrient-competitive microenvironment of ectopic lesions, insufficient glucose uptake restricts ATP production in NK cells, which impairs the biosynthesis and exocytosis of cytotoxic molecules including granzyme and perforin, blocks the formation of effective cytotoxic synapses with ESCs, and ultimately disrupts cytotoxic signaling.^142^ Besides, metabolic products of energy stress can also impair NK cell cytotoxicity. Excessive lactate accumulation activates the AMPK signaling pathway which significantly downregulates the expression of cytotoxicity-associated receptors such as CD16 and killer cell lectin like receptor K1 (KLRK1), directly hinders the recognition and binding of NK cells and inhibits the initiation of cytotoxicity.^143^^,^^144^ Meanwhile, high expression of IDO1 in ESCs activates the tryptophan-kynurenine pathway which markedly suppresses the mTOR pathway, reduces the production of the cytotoxicity-related molecule interferon γ (IFN-γ) and further impairs the cytotoxic capacity of NK cells.^145^ Furthermore, energy stress-induced macrophage M2 polarization leads to the continuous release of abundant IL-10 and TGF-β which downregulate cytotoxicity-associated receptor expression, inhibit cytotoxic synapse formation and cytotoxic molecule production, and thus markedly impair the ability of NK cells to eliminate ESCs.^146^^,^^147^

#### Metabolic regulation of neutrophil extracellular traps

Studies have shown that neutrophils are significantly elevated in the blood, ascites and ectopic lesions of EMs patients.^148^^,^^149^ Metabolic reprogramming of neutrophils in response to energy stress in ectopic lesions induces excessive neutrophil extracellular traps (NETs) formation and defective clearance, thereby facilitating lesion implantation, chronic inflammation, tissue damage, and immune escape in EMs.^150^ Hypoxia in ectopic lesions activates the HIF-1α signaling pathway which drives glycolysis to supply sufficient ATP for NETs formation. PKM2 nuclear translocation activates HMGB1, which then binds to toll-like receptor 4 (TLR4) on the neutrophil surface and induces the expression of NET-related genes. Meanwhile, massive lactate production enhances the activity of peptidyl arginine deiminase 4 (PADI4) via G-protein coupled receptor 81 (GPR81) to promote chromatin decondensation and NETs release. And decreased activities of isocitrate dehydrogenase (IDH) and succinate dehydrogenase (SDH) cause substantial succinate accumulation, which activates PADI4 and amplifies ROS signaling to induce NETs formation.^151^^,^^152^ After excessive NETs formation, their mesh-like structures serve as physical scaffolds to capture free ESCs in the ectopic microenvironment and promote cells adhesion, colonization, and invasion. Concurrently, NETs release abundant pro-inflammatory factors, such as IL-1β and TNF-α, VEGF, cytotoxic proteins and proteases to directly damage peritoneal mesothelial cells, trigger chronic inflammation, and aggravate tissue injury. In addition, NETs can release large amounts of anti-inflammatory factors including TGF-β, IL-4, and IL-10, suppress the cytotoxic function of T cells and NK cells and promote macrophage M2 polarization, thereby facilitating immune escape of ESCs.^153^^,^^154^^,^^155^

#### Metabolic basis of T cell imbalance

As key executors of adaptive immunity, functional imbalance of T lymphocytes is a critical basis for immune microenvironment disturbance in EMs. Energy stress-induced CTL exhaustion, Th1/Th2 deviation, and Th17/Treg imbalance are core mechanisms that drive immune escape, lesion survival and disease progression.^156^^,^^157^ Severe hypoxia and nutrient competition in ectopic lesions drive infiltrating T cells to undergo functional metabolic reprogramming. Glycolysis-dependent effector T cells, including CTL, Th1, and Th17, exhibit impaired function due to energy crisis, while the Treg cells that adapt to OXPHOS and are adept at utilizing fatty acid oxidation expand significantly and exert immunosuppressive effects.^158^^,^^159^ The HIF-1α/lactate axis depletes glucose and generates large amounts of lactate, which directly inhibits the activity of sodium-hydrogen exchanger 1 (NHE1) on the CTL cell membrane, reducing perforin and granzyme secretion to impair CTL cytotoxicity, as well as inhibits T cell receptor signaling and the mTOR pathway to further limit effector T cell proliferation and cytokine secretion.^160^^,^^161^ Notably, accumulated lactate can promote CD4^+^ T cells differentiation into Tregs via G-protein coupled receptor 81 (GPR81), and stabilize HIF-1α to drive the generation of Th17 cells and the secretion of abundant pro-inflammatory factors, finally forming a microenvironment with coexisting immunosuppression and chronic inflammation.^162^ In addition, highly expressed IDO1 in ectopic lesions catalyzes tryptophan degradation, causing cell-cycle arrest and functional damage of effector T cells.^163^^,^^164^ Simultaneously, massive accumulation of its toxic metabolite kynurenine activates the aryl hydrocarbon receptor (AhR) to drive naive CD4^+^ T cell differentiation into immunosuppressive Tregs and suppress pro-inflammatory Th17 cell formation, thereby constraining excessive inflammatory damage and establishing a local microenvironment of immune escape and tolerance that supports the survival of ectopic endometrial tissue.^165^^,^^166^

#### Metabolic adaptability of B cells

**Figure 4:**
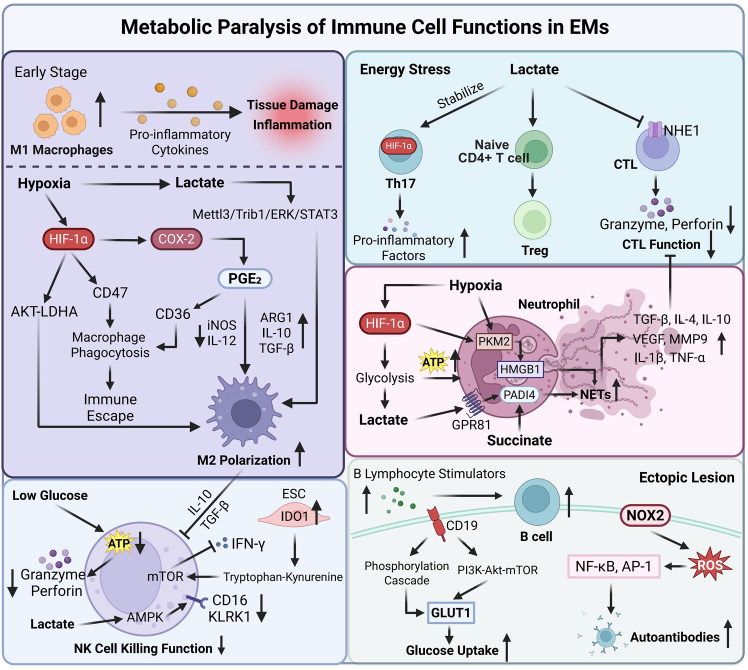
Energy stress-induced adaptive functional alterations of immune cells in EMs In the metabolic stress microenvironment of EMs, immune cells exhibit adaptive functional alterations: (1) M2 polarization of macrophages; (2) impaired cytotoxic activity of NK cells; (3) NETs formation of neutrophils; (4) exhaustion of CTL function with increased generation of Tregs; (5) marked infiltration of B lymphocytes. These adaptive immune cell alterations collectively construct an immunosuppressive microenvironment in EMs, driving the survival, and progression of ectopic lesions.

Studies have revealed that B cell number and activity are significantly increased in the peritoneal cavity and peripheral blood of EMs patients. The serum and local microenvironment of ectopic lesions in these patients have significantly higher levels of B lymphocyte stimulator, which causes massive plasma cell infiltration and intensifies autoimmune responses.^167^^,^^168^ B cell metabolic adaptability is primarily mediated by the CD19 signaling pathway and the oxidative stress pathway mediated by NADPH oxidase 2 (NOX2). The CD19 co-stimulatory signal on B cell surfaces promotes GLUT1 translocation to the cell membrane via phosphorylation cascades and upregulates GLUT1 expression through the PI3K-Akt-mTOR pathway, both of which markedly enhance B cell glucose uptake capacity.^169^ Meanwhile, sustained oxidative stress activates the NOX2 pathway to produce abundant ROS as secondary messengers. These ROS promote B cell differentiation into plasma cells and cause massive autoantibody production through important molecules like NF-κB and activator protein-1 (AP-1), exacerbating tissue damage and local inflammation.^170^^,^^171^ The disruption of immune homeostasis caused by this pathological metabolic and functional remodeling of B cells accelerates the pathological development of EMs (Figure 4).

### Metabolic expression of immune checkpoints (ICPs) in EMs

#### Hypoxia-lactate-lipid axis drives PD-1/PD-L1 upregulation

Programmed cell death protein 1 (PD-1) and its ligand programmed cell death ligand 1 (PD-L1) are key checkpoint molecules mediating immunosuppression, which impair the activity and function of effector T cells and enhance Treg-mediated immune tolerance.^172^^,^^173^ The expression levels of molecules associated with PD-1/PD-L1 pathway are significantly increased in the serum, ectopic endometrial tissues, and peritoneal fluid of EMs patients. These molecules facilitate the establishment of an immunosuppressive microenvironment that supports the colonization and survival of ectopic endometrium. Additionally, this pathway is also reported to be closely linked to the progression of EMs-associated ovarian cancer and the occurrence of EMs-related infertility.^174^^,^^175^ Within the energy-stressed microenvironment of EMs, hypoxia stabilizes HIF-1α to directly bind to the promoter region of *CD274*, the gene encoding PD-L1, and initiate PD-L1 transcription, while also promoting VEGF secretion to indirectly induce PD-L1 expression in immune cells.^176^^,^^177^ Concurrently, accumulated lactate triggers the cAMP signaling pathway via GPR81 to boost PD-L1 transcription and alters CD274 chromatin conformation through histone lactylation to maintain PD-L1 expression.^178^ Furthermore, PPAR-γ signaling is activated by bioactive lipids derived from local lipid metabolic disorders, which causes a significant upregulation of PD-L1 expression on immune cells, including macrophages. Notably, energy deprivation can further trigger the AMPK/mTOR signaling pathway, enhancing cellular sensitivity to PD-1/PD-L1 signals and thus amplifying the immunosuppressive effect.^179^ The hypoxia-lactate-lipid axis synergistically drives the aberrant upregulation of the PD-1/PD-L1 pathway, which constitutes the core mechanism of immunosuppression in EMs and acts as a key factor promoting immune escape of ectopic lesions.

#### Dysregulated amino acid metabolism induces CTLA-4 overexpression

Cytotoxic T-lymphocyte-associated antigen 4 (CTLA-4), a pivotal receptor governing the negative regulation of effector T cell activation and the enhancement of Treg immunosuppressive function, has been reported to be closely associated with advanced EMs progression and EMs-related infertility.^180^ Studies have demonstrated that aberrant CTLA-4 elevation in the peritoneal fluid of EMs patients is strongly linked to local amino acid metabolic dysfunction.^181^^,^^182^ In the ectopic microenvironment, energy stress induces high expression of IDO in ESCs mediating tryptophan catabolism and kynurenine buildup, which subsequently activates AhR signaling pathway to drive naive T cell differentiation into Tregs with elevated CTLA-4 expression and further amplifies the suppression of effector T cells.^183^ Meanwhile, the aberrant overexpression of ARG1 efficiently degrades arginine in the ectopic milieu, depriving effector T cells of the key substrate essential for proliferation and promoting their conversion into fatty acid oxidation-dependent Tregs, thereby sustaining stable CTLA-4 overexpression and a prolonged immunosuppressive effect.^184^ In addition, ESCs exposed to energy stress secrete substantial TGF-β, which initiates Smad2/3 pathway activation in Tregs, whereupon the activated Smad complex directly engages Smad-binding motifs in the CTLA-4 promoter to upregulate CTLA-4 transcription and expression, thereby reinforcing the immunosuppressive niche and propelling EMs pathogenesis.^185^^,^^186^

#### Energy deprivation and oxidative stress induce TIM-3/Gal-9 upregulation

In EMs, there is persistent activation of the T cell immunoglobulin and mucin domain-containing protein 3/galectin-9 (TIM-3/Gal-9) signaling pathway, along with dysregulation of its pathway-dependent regulatory functions.^187^^,^^188^ Studies have revealed that TIM-3 expression is significantly upregulated in the peripheral blood, peritoneal fluid and ectopic tissues of EMs patients, which is strongly associated with ectopic lesion colonization and local inflammation persistence. Peripheral blood Tregs and CD4^+^ T cells exhibit significantly elevated surface Gal-9 expression, and this upregulation trend extends to NK cells and all T cell subsets present in the peritoneal fluid.^189^^,^^190^ The expression of TIM-3/Gal-9 has also been reported to be closely associated with cellular metabolic status. Hypoxia directly drives Gal-9 transcription via the HIF-1α signaling pathway, and accumulated lactate can upregulate TIM-3 expression on macrophages and T cells through signaling pathways such as cAMP. Elevated ROS levels can trigger the activation of inflammatory signaling pathways including NF-κB, which in turn drives TIM-3 transcription and expression.^191^^,^^192^^,^^193^^,^^194^^,^^195^ Subsequently, highly expressed TIM-3/Gal-9 not only impairs CD4^+^/CD8^+^ T cell function and exacerbates their exhaustion but also diminishes NK cell activity and triggers macrophage M2 polarization, collectively establishing an ectopic immunosuppressive microenvironment that promotes EMs progression and malignant transformation.^196^^,^^197^^,^^198^

#### Hypoxia-glycolysis promotes the expression of the CD47/SIRPα signaling pathway in macrophage

**Figure 5:**
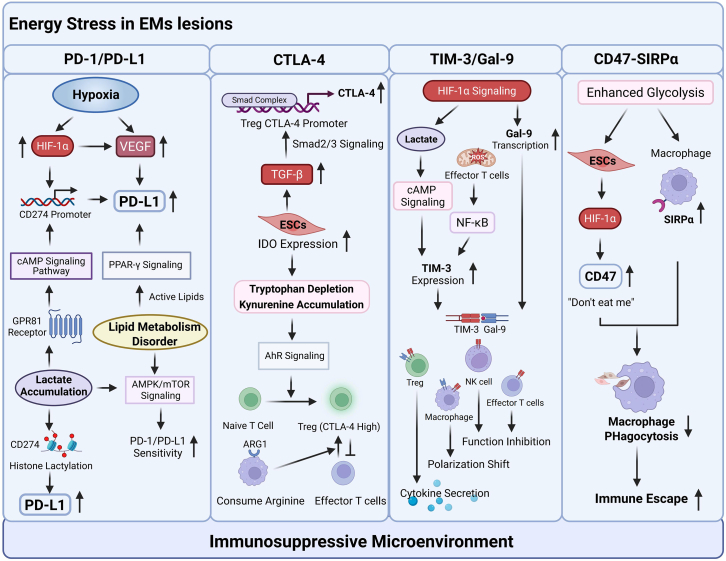
Metabolic regulation of ICPs promoting immune escape in EMs The metabolic microenvironment of EMs (including hypoxia, lactate accumulation, and metabolic disorders) drives abnormal expression of key ICPs to form an immunosuppressive network: (1) hypoxia-lactate-lipid axis upregulates PD-1/PD-L1; (2) amino acid metabolism disorders and TGF-β/Smad pathway induce high CTLA-4 expression; (3) lactate accumulation and increased ROS upregulate TIM-3/Gal-9; (4) hypoxia-glycolysis promotes CD47/SIRPα signaling. All above inhibit effector T cells, regulate immune cell functions, mediate immune escape of ectopic lesions, and drive EMs progression.

It has been reported that CD47, which is highly expressed in ectopic lesions, acts as a “don’t eat me” signal that binds to signal-regulated protein α (SIRPα) on the surface of macrophages, mediating immune escape of ectopic lesions by inhibiting macrophage phagocytosis.^199^ Under the energy-stressed microenvironment of ectopic lesions, enhanced glycolysis and activation of the HIF-1α signaling pathway significantly upregulate CD47 expression levels, and they simultaneously induce M2 polarization of macrophages and promote SIRPα expression.^135^^,^^200^ Moreover, abundant lactate can strengthen signal transduction efficiency, amplify immunosuppressive effects, and synergize with immune checkpoints like PD-1/PD-L1 to form an immunosuppressive network, ultimately accelerating EMs progression (Figure 5).^201^^,^^202^

## Therapeutic strategies and clinical translation targeting metabolism-immunity in EMs

### Advances and exploration of therapeutic strategies targeting metabolic regulatory pathways

Beyond the pathway-based mechanistic interpretations, emerging human single-cell transcriptomic studies have provided critical evidence supporting the metabolic-immune regulation in EMs, identifying distinct senescence-associated phenotypes and impaired decidualization in patient-derived endometrial tissues that are closely linked to metabolic stress, mitochondrial dysfunction, and downstream immune dysregulation.^23^^,^^203^^,^^204^ These findings offer important support for further exploration of therapeutic strategies targeting the metabolic-immune axis in EMs and their clinical translation. At present, metabolic pathway-targeted inhibitors and modulators have made steady progress in preclinical and early clinical studies, showing promising therapeutic potential for EMs.

#### Current research advances and application prospects of glycometabolism inhibitors

As a core transcription factor mediating the adaptation of ESCs to hypoxic microenvironments, HIF-1α acts as a critical driver of pathological progression in EMs and has emerged as a key therapeutic target.^84^ At the mechanistic level, an *in vitro* study has confirmed that romidepsin, an inhibitor of histone deacetylase (HDAC), can suppress the expression of HIF-1α in ESCs, reduce the transcription and secretion of VEGF, and thereby inhibit the angiogenesis process of EMs.^205^ Another study has reported that 2-methoxyestradiol, a systemic angiogenesis inhibitor, can exert anti-lesion effects on EMs by suppressing HIF-1α expression and downregulating transcription of VEGF, phosphoglycerate kinase (PGK) and GLUT1.^206^ Besides, traditional Chinese medicine formulations, such as Luoshi Neiyi Recipe, Wenshen Xiaozheng Decoction, and Guizhi Fuling Capsule, have also demonstrated inhibiting the proliferation of ESCs and reducing the volume of ectopic lesions by targeting the HIF-1α signaling pathway.^207^^,^^208^^,^^209^ Beyond the HIF-1α-targeted therapies, key glycolytic enzymes such as PDK, LDHA, and PKM2 have also been identified as vital therapeutic targets for EMs.^210^ Lee et al. demonstrated that dichloroacetate (DCA) inhibits PDK1 activity to reduce lactate production and oxygen consumption as well as to induce apoptosis in ESCs.^88^ In a separate *in vivo* study, Horne AW et al. found that oral administration of DCA decreased lactate concentrations and reduced the size of ectopic lesions in a murine model of EMs.^211^ And the herbal extract of *Prunella vulgaris* has been reported to promote apoptosis of ESCs and suppress the growth of ectopic lesions via targeting LDHA and PDK1/3^212^. Additionally, Yao et al. found that cinnamic acid from the medicinal plant *Cinnamomum cassia* exerts an anti-EMs effect by targeting PKM2 to suppress the survival and migration of ESCs.^212^ Although numerous studies have confirmed that glycolysis and its key molecules contribute to EMs progression, clinical studies on glycolysis inhibitors for EMs treatment remain relatively scarce, with most limited to preliminary validation of single-target efficacy. Therefore, extensive systematic and multi-dimensional in-depth studies are urgently needed in the future to provide a solid scientific basis for the translation of glycolysis inhibitors into clinical therapy for EMs.

#### Synergistic effects and research progress of lipid metabolism modulators

Studies have confirmed that lipid metabolites, including phosphatidic acid, phosphatidylcholine, and phosphatidylserine, hold promise as potential biomarkers for the early diagnosis of EMs.^38^ As shown by *in vitro* and *in vivo* studies, statins such as atorvastatin and pravastatin target 3-hydroxy-3-methylglutaryl-coenzyme A (HMG-CoA) reductase to inhibit ESCs proliferation and ectopic lesion growth, enhance the local immune microenvironment and preserve ovarian reserve and fertility.^213^^,^^214^ Similarly, PRGL493, an inhibitor of long-chain acyl-CoA synthetase 4 (ACSL4), has been shown to enhance endometrial receptivity, providing a potential targeted intervention strategy for EMs-associated infertility.^215^ PPARγ agonists, including rosiglitazone and pioglitazone, have been reported to significantly reduce the number and size of ectopic endometrial lesions, inhibit inflammatory infiltration in the local microenvironment, and preserve patient fertility.^216^^,^^217^^,^^218^ Resveratrol, a naturally occurring substance, has been demonstrated in animal experiment to activate the PPARγ signaling pathway to control lipid metabolism and macrophage polarization in a rat EMs model.^219^ Additionally, phase IV clinical trials have validated resveratrol’s safety, efficacy, and analgesic properties in patients with EMs. Rofecoxib, a selective COX-2 inhibitor, has been shown to exert notable therapeutic effects against pain associated with EMs, offering a targeted option for symptom relief in EMs patients.^220^ Nevertheless, potential adverse effects like lipid metabolism disorders and impaired hepatorenal function continue to pose a challenge to the clinical translation of lipid metabolism regulators for EMs. Thus, further preclinical research and the development of lesion-targeted therapeutics are required to increase local efficacy and minimize side effects.

#### Potential and exploratory research of amino acid levels as biomarkers

Although many studies have shown that the glutamine metabolism pathway and targets, such as IDO1 and TDO, are critical for the development of EMs and have therapeutic potential, their underlying mechanisms remain unclear.^44^^,^^221^ The ratio of circulating proline to glutamine is markedly increased in EMs patients, according to metabolomic studies based on nuclear magnetic resonance (NMR), indicating that these two non-essential amino acids have the potential to serve as non-invasive biomarkers for EMs diagnosis and prognostic evaluation.^43^ An observational paired study involving women with ovarian EMs demonstrated that the mean diameter of ovarian endometriomas was significantly reduced in patients treated with N-acetylcysteine (NAC), which was attributed to the ability of NAC to increase intracellular cysteine levels, thereby exerting antioxidant and free radical scavenging effects.^222^ Metabolomic analyses have shown that concentrations of aspartic acid, histidine, and glycine are significantly elevated in EMs patients, while those of tyrosine, alanine and other amino acids are markedly reduced, a change potentially linked to glucose metabolism and oxidative stress in ectopic lesions.^223^^,^^224^ These distinctive amino acid profiles show promise as potential biomarkers for disease diagnosis and provide important insights into the pathophysiology of EMs.

#### Application progress and transformation potential of mitochondrial function modulators

Coenzyme Q10 (CoQ10), a critical component of the mitochondrial electron transport chain, is significantly decreased in EMs patients, and its supplementation can markedly shrink ectopic lesion volume and ameliorate peritoneal adhesions in rats.^53^ Multiple *in vitro* and *in vivo* studies have confirmed that melatonin can penetrate mitochondrial membranes, disrupt the redox status of ESCs and inhibit lipid peroxidation.^225^^,^^226^^,^^227^ A clinical controlled trial has demonstrated that melatonin effectively alleviates EMs-associated chronic pelvic pain and improves patient quality of life.^228^ Additionally, methyl 3,4-dihydroxybenzoate (MDHB), a natural plant extract, improves mitochondrial function, increases mitochondrial membrane potential, and upregulates genes linked to antioxidant stress, all of which have positive effects on infertility caused by EMs.^229^ The dietary flavonoid quercetin induces apoptosis of ESCs via mitochondrial membrane potential loss and ROS generation, and clinical studies have assessed its prognostic value in EMs patients to provide evidence for clinical translation.^230^^,^^231^^,^^232^ Furthermore, a variety of plant extracts, such as curcumin, puerarin, ginsenosides, soy isoflavones, naringenin, and aesculetin, are promising therapeutic candidates because they can prevent the progression of EMs by controlling mitochondrial function.^233^^,^^234^^,^^235^^,^^236^ These results demonstrate the value of focusing on mitochondrial redox and metabolic pathways to create innovative EMs treatments, yet their translational potential necessitates additional validation via thorough clinical trials (Table 1).

**Table 1:** Therapeutic evidence for metabolic-targeted interventions in EMs

Evidence Level	Study Type	Metabolic Target	Inhibitor/Agent	Expected Effect
Preclinical	*in vitro*	HIF-1α	romidepsin^205^	suppress HIF-1α expression, reduce VEGF transcription and secretion in ESCs
Preclinical	*in vitro*	HIF-1α	2-Methoxyestradiol^206^	suppress HIF-1α expression, downregulate the transcription of VEGF, PGK and GLUT1
Preclinical	*in vitro* & *in vivo*	HIF-1α	Luoshi Neiyi recipe, Wenshen Xiaozheng decoction, Guizhi Fuling capsule^207^^,^^208^^,^^209^	inhibit ESCs proliferation and reduce ectopic lesion volume
Preclinical	*in vitro*	lactate	DCA^88^	reduce lactate production and oxygen consumption, induce apoptosis in ESCs
Preclinical	*in vivo*	PDK	DCA^211^	reduce lactate production and size of ectopic lesions
Preclinical	*in vitro* & *in vivo*	LDHA and PDK1/3	Prunella vulgaris extract^237^	promote ESCs apoptosis and suppress ectopic lesion growth
Preclinical	*in vitro*	PKM2	cinnamic acid^212^	suppress ESCs survival and migration
Preclinical	*in vitro* & *in vivo*	HMG-CoA reductase	atorvastatin, pravastatin^213^^,^^214^	inhibit ESCs proliferation and ectopic lesion growth, and preserve ovarian reserve
Preclinical	*in vitro* & *in vivo*	ACSL4	PRGL493^215^	enhance endometrial receptivity
Preclinical	*in vitro* & *in vivo*	PPARγ	rosiglitazone, pioglitazone^216^^,^^217^^,^^218^	reduce lesion number and size, inhibit inflammation, preserve fertility
Preclinical	*in vivo*	PPARγ	resveratrol^219^	control lipid metabolism, regulate macrophage polarization in a rat EMs model
Clinical	phase IV clinical trial	PPARγ	resveratrol	reduce EMs-related pain
Clinical	clinical study	COX-2	rofecoxib^220^	alleviate pain
Preclinical	*in vitro* & *in vivo*	IDO1, TDO^221^	/	modulate glutamine metabolism
Clinical	observational	proline/glutamine ratio^43^	/	serve as non-invasive diagnostic biomarker
Clinical	observational paired study	cysteine	NAC^222^	antioxidant and free radical-scavenging effects
Preclinical	*in vitro* & *in vivo*	mitochondrial electron transport chain	CoQ10^53^	maintain redox balance, restore mitochondrial function
Preclinical	*in vitro* & *in vivo*	mitochondrial membrane	melatonin^225^^,^^226^^,^^227^	disrupt ESCs redox status, inhibit lipid peroxidation
Clinical	clinical controlled trial	mitochondrial membrane	melatonin^228^	alleviate chronic pelvic pain
Preclinical	*in vitro* & *in vivo*	mitochondrial function	MDHB^229^	improve pregnancy outcomes for EMs-associated infertility
Preclinical	*in vitro* & clinical	mitochondrial membrane potential	Quercetin^230^^,^^231^^,^^232^	induce ESC apoptosis
Preclinical	*in vitro* & *in vivo*	mitochondrial function	curcumin, puerarin, ginsenosides, soy isoflavones, naringenin, aesculetin^233^^,^^234^^,^^235^^,^^236^	control mitochondrial function, prevent EMs progression

### Synergistic strategies and development prospects of metabolic-immune combined intervention

The complex metabolic-immune regulatory network not only promotes the survival and immune evasion of ectopic lesions but also directly impairs endometrial homeostasis, decidualization, and implantation, thereby compromising reproductive function and ultimately leading to infertility in EMs patients. Therefore, the therapeutic paradigm for EMs must shift from monotherapies of hormonal modulation or isolated target intervention to an era of integrative medicine characterized by metabolic-immune combined intervention and systemic remodeling of the endometrial microenvironment. Currently, a growing number of studies have focused on the identification and exploration of targets for combined therapy. Wang et al. confirmed that ubiquitin-conjugating enzyme E2S (UBE2S) facilitates EMs progression by regulating glucose metabolic reprogramming and the immune microenvironment, and its inhibitor cephalomannine downregulates GLUT1 expression to suppress glycolysis in ESCs, blocks M2 macrophage polarization, and ultimately alleviates lesion fibrosis.^238^ Another study indicated that targeting the PDPK1-CD47-LDHA signaling axis can simultaneously inhibit glycolysis and M2 macrophage polarization during EMs progression.^135^ Notably, ovarian clear cell carcinoma has been reported to be closely associated with EMs. An *in vivo* animal study revealed that the combination therapy of histone deacetylase 6 (HDAC6) inhibitor ACY1215 and anti-PD-L1 treatment can suppress the progression of ARID1A-mutant ovarian clear cell carcinoma by enhancing the activity of CTLs.^239^

However, there is still a dearth of comprehensive clinical research on targeting the energy stress-mediated metabolic-immune axis. Future studies should rely on cutting-edge technologies including multi-omics integrative analysis and immune cell mapping to further decipher the metabolic-immune network in EMs and improve the scientific rigor of target screening and combination regimen design. The development of EMs combination regimens should be supported both theoretically and practically by mechanism studies and clinical trials carried out concurrently. Moreover, in order to balance the long-term enhancement of the ectopic microenvironment by metabolic inhibitors and the maximum effectiveness of immune inhibitors during crucial therapeutic windows, the ideal dosage of combination regimens should be improved through preclinical and clinical research to improve safety and efficacy.

## Conclusion and perspectives

This review establishes a unified regulatory cascade connecting intrinsic genetic mutations, microenvironmental energy stress, metabolic remodeling, and immune evasion in EMs, provides new mechanistic perspectives on EMs pathological progression, and offers promising directions for the development of combined metabolic-immune therapeutic strategies.

Nevertheless, current studies on mutation-driven metabolic reprogramming, especially its functional role in EMs, remain relatively scarce. Drawing on the well-established paradigm of tumor metabolic research, where oncogenic mutation-mediated constitutive metabolic reprogramming engages in tight interplay with microenvironmental energy stress-elicited passive metabolic adaptation to form a bidirectional self-reinforcing loop,^240^^,^^241^^,^^242^ we propose that future investigations in EMs should prioritize the following three critical directions aligned with the dual-driver model: (1) Clarify the core molecular mechanisms underlying oncogenic mutation-induced active metabolic reprogramming in EMs; (2) systematically elucidate the dynamic interaction patterns between intrinsic genetic alterations and extrinsic microenvironmental energy stress; (3) uncover the bidirectional reinforcing loop between these two driving axes that fuels EMs progression.

Elucidation of this bidirectional regulatory network will help uncover the pathological essence of metabolic disorder and immune dysfunctions in EMs, and provide a novel theoretical basis and potential targeted therapeutic strategies for clinical intervention and disease blockade.

## Acknowledgments

This work was supported by grants to Jing Sun from the 10.13039/501100003399Science and Technology Commission of Shanghai Municipality (22Y11906100) and Shanghai Outstanding Academic Leaders Plan to Jing Sun (Year 2019).

## Author contributions

P.Y. and T.W. contributed equally to this study, conceived the idea, drafted the main manuscript and prepared the figures. W.L. and Y.M. conducted the literature search. Y.Z. and J.S. revised the review. All authors reviewed and approved the final version of the manuscript.

## Declaration of interests

The authors have declared that no competing interests exist.
